# ‘Broken symmetries’ in macromolecular crystallography: phasing from unmerged data

**DOI:** 10.1107/S0907444909053578

**Published:** 2010-03-24

**Authors:** Marc Schiltz, Gérard Bricogne

**Affiliations:** a École Polytechnique Fédérale de Lausanne (EPFL), Laboratoire de Cristallographie, CH-1015 Lausanne, Switzerland; b Global Phasing Ltd, Sheraton House, Castle Park, Cambridge CB3 0AX, England

**Keywords:** broken symmetry, phasing, radiation damage, polarization anisotropy

## Abstract

The space-group symmetry of a crystal structure imposes a point-group symmetry on its diffraction pattern, giving rise to so-called symmetry-equivalent reflections. Instances in macromolecular crystallography are discussed in which the sym­metry in reciprocal space is broken, *i.e.* where symmetry-related reflections are no longer equivalent. Such a situation occurs when the sample suffers from site-specific radiation damage during the X-ray measurements. Another example of broken symmetry arises from the polarization anisotropy of anomalous scattering. In these cases, the genuine intensity differences between symmetry-related reflections can be exploited to yield phase information in the structure-solution process. In this approach, the usual separation of the data merging and phasing steps is abandoned. The data are kept unmerged down to the Harker construction, where the symmetry-breaking effects are explicitly modelled and refined and become a source of supplementary phase information.

## Abstract 2

Site-specific radiation damage and anisotropy of anomalous scattering can induce intensity differences in symmetry-related reflections. If the data are kept unmerged, these symmetry-breaking effects can become a source of phase information.

## 1. Introduction

The space-group symmetry of a crystal structure generally imposes a point-group symmetry in reciprocal space, giving rise to so-called symmetry-equivalent reflections. This usually introduces a certain redundancy in diffraction data recorded with an area detector and is exploited in X-ray crystallo­graphy to increase the accuracy of the data by averaging over the symmetry-equivalent measurements (data merging). How­ever, genuine intensity differences between symmetry-related reflections can occur when the sample suffers from site-specific radiation-induced changes during X-ray data collection (Schiltz *et al.*, 2004 ▶; Schiltz & Bricogne, 2007 ▶) or in the presence of the rather obscure phenomenon of anisotropy of anomalous scattering (AAS; Bricogne *et al.*, 2005 ▶; Sanishvili *et al.*, 2007 ▶; Schiltz & Bricogne, 2008 ▶). In such cases, the use of unmerged data can generate phasing power through the intensity differences of symmetry-related reflections.

In the present communication, we will review the symmetry-breaking effects in reciprocal space which arise from site-specific radiation damage and AAS. We start with a brief introduction to experimental phasing methods and show how the effects of site-specific radiation damage and AAS can be incorporated into the general scheme of experimental phasing methods using an extended Harker construction. In line with the general philosophy of contributions to the CCP4 Study Weekend, this is intended to be a ‘tutorial’ paper, in which formal developments are kept to a minimum. The presentation given here is intended to serve as an introduction and guide to the more comprehensive papers that we have published on this subject.

## 2. Basics of experimental phasing methods

### 2.1. Experimental phasing viewed as an interference experiment

**Figure 1:**
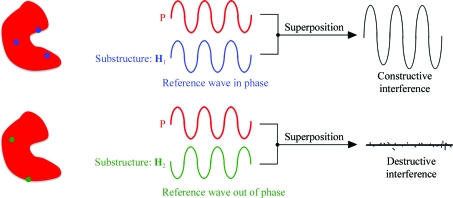
Experimental phasing viewed as an interference experiment. The X-rays scattered by a subset of atoms (the substructure) provide a reference wave of known phase and amplitude which interferes with the wave scattered from the unknown structure (in red). Depending on the relative phase difference between these two waves, the superposition will yield partially constructive or destructive interference effects which produce measurable intensity modulations.

Experimental phasing methods are, in their bare essence, based on interference effects between the wave scattered from the unknown structure and the wave scattered from a set of atoms whose positions are, at least approximately, known. This is called the *substructure* (Fig. 1 ▶). The X-rays scattered by the substructure provide a reference wave of known phase and amplitude which interferes with the wave scattered from the unknown structure. Depending on the relative phase difference between these two waves, the superposition will yield partially constructive or destructive interference effects which produce measurable intensity modulations. In this way, information about the unknown (and not directly observable) phases is transferred to measurable intensity variations.

**Figure 2:**
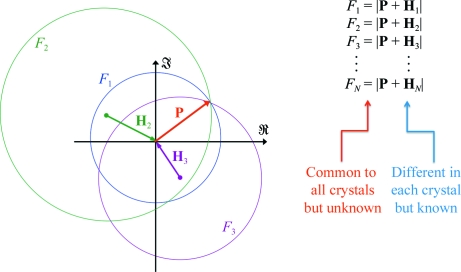
The Harker construction. In the example given the first ‘derivative’ is a native crystal, so that **H**_1_ = 0.

Formally, for a given reflection **h**, let us denote the structure amplitude of the wave scattered from the unknown part by **P**(**h**) = *P*(**h**)exp[*i*ϕ_*P*_(**h**)] and the structure amplitude of the reference wave, scattered from the substructure, by **H**(**h**) = *H*(**h**)exp[*i*ϕ_*H*_(**h**)]. Their superposition then yields for the structure amplitude of the diffracted wave
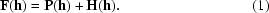
Its square modulus, which is proportional to the diffracted intensity, yields an expression that contains an interference term which depends on the relative phase difference ϕ_*P*_(**h**) − ϕ_*H*_(**h**), 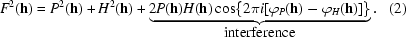
Even if the complex amplitude of the reference wave, **H**(**h**), is known, a single measurement *F* ^2^(**h**) will not yield all the information about the unknown structure since there are two unknowns [*P*(**h**) and ϕ*_P_*(**h**)] in the above equation. An additional ambiguity arises for acentric reflections since the unknown phase, ϕ*_P_*(**h**), is only defined through its absolute difference with respect to the phase of the reference wave, ϕ*_H_*(**h**). It is therefore required to have some means of modifying the reference wave and then recording a series of *N*_**h**_ measurements, 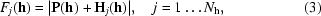
each with a different reference wave **H**_*j*_(**h**). Putting it differently, the complex structure factor, **F**(**h**), is split up into an unknown part, **P**(**h**), which is constant, and a known part, **H**(**h**), which is varied between different measurements. Each equation (3) defines a circle on the complex plane with radius *F*_*j*_(**h**) and centre −**H**_*j*_(**h**). The set of all such *N*_**h**_ circles intersect at the point **P**(**h**) and represents what is known as the *Harker construction* (Harker, 1956 ▶; Fig. 2 ▶).

### 2.2. The method of isomorphous replacement

In the method of isomorphous replacement (Green *et al.*, 1954 ▶; Harker, 1956 ▶), the variations in the reference wave **H**(**h**) are achieved by collecting diffraction data from several crystals (different *derivatives*) containing different substructures (usually consisting of heavier atoms). In modern treatments of this method a native crystal has no special status and is simply considered as a derivative for which **H**(**h**) = 0 (de La Fortelle & Bricogne, 1997 ▶). The standard radiation-induced phasing (RIP) method (Ravelli *et al.*, 2003 ▶; Nanao & Ravelli, 2006 ▶), in which a diffraction data set is first collected from a fresh crystal followed by exposure to X-ray or UV radiation and subsequent collection of a second data set containing site-specific radiation damage, can be subsumed under the category of isomorphous replacement.

In these cases, the index *j* labels the various derivatives. For success of the method, it is important that the constant part, whose structure factor is **P**(**h**), remains essentially un­altered in the different derivatives: a condition known as isomorphism. In other words, the variations in the diffracted intensities must solely arise from modulations of the substructure.

### 2.3. Multi-wavelength methods

**Figure 3:**
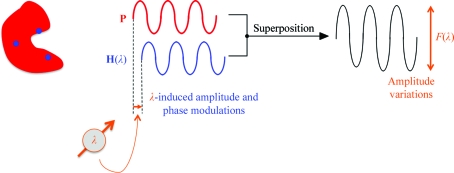
In the multi-wavelength (MAD) method the reference wave is modulated in phase and amplitude by exploiting the wavelength-dependence of the atomic scattering factors *f*′ and *f*′′ of those atoms that constitute the substructure. The wavelength-dependent variations of the wave scattered from the substructure will, after superposition with the wave scattered from the unknown (and wavelength-independent) part of the structure, give rise to measurable amplitude variations.

In the multi-wavelength anomalous diffraction (MAD) method (Hendrickson, 1991 ▶), the reference wave is modulated by exploiting the wavelength-dependence of the atomic scattering factors *f*′ and *f*′′ in the vicinity of an absorption edge of those atoms that constitute the substructure (Fig. 3 ▶). In this case the index *j* labels the various data sets recorded at different wavelengths. Again, it is important that the constant part **P**(**h**) remains essentially unaffected by the wavelength changes.

## 3. Phasing from unmerged data

In the above examples the label *j* refers to a *data set*, *e.g.* a set of reflections recorded on a particular isomorphous derivative or a set of reflections recorded at a particular wavelength. It is often assumed that the set of all structure-factor amplitudes *F*_*j*_(**h**) which share the same label *j* would need to form a coherent data set related to a particular crystal structure. However, this is not a necessary requirement since the Harker construction (3) is set up for each individual reflection **h**. The index *j* is really just a generic label which encodes book-keeping information about the experimental parameters that selectively modulate the reference wave **H** *_j_*(**h**). It is therefore equally possible to set up a Harker construction with several measurements *F*_*j*_(**h**) of the same or of a symmetry-related reflection all recorded on the same sample and at the same wavelength. It is, however, necessary that these data are affected in one way or another by specific variations in the substructure amplitude **H** *_j_*(**h**). The generalized Harker construction for symmetry-related measurements is discussed in more detail in Appendix *A* [App appa].

In many circumstances, variations in the intensities of symmetry-related reflections or of repeated measurements of the same reflection simply arise from geometric or experimental factors such as differences in absorption, irradiated crystal volume, incident beam flux or decay owing to overall (nonspecific) radiation damage. These variations do not specifically affect the substructure and therefore do not produce differential modulations which are exploitable for phase determination *via* the Harker construction. They can be corrected for by multiplicative (scale) factors which are usually determined empirically and applied to the measured intensities. Since such intensity variations cannot generate phase information, the various symmetry-related intensity measurements and repeated measurements of the same reflection are usually merged into a single structure-factor amplitude after the correction factors have been applied.

**Figure 4:**
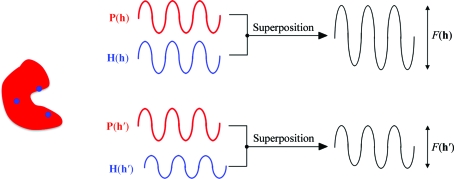
Phasing through symmetry-breaking effects. If the reflections **h** and **h**′ are symmetry-related, the wave scattered by the unknown part of the structure [**P**(**h**)] is identical (possibly up to a fixed phase shift; see Appendix *A* [App appa]) for both reflections. However, symmetry-breaking effects that specifically affect the substructure will give rise to measurable amplitude differences between symmetry-related reflections.

However, there are several instances in macromolecular crystallography where symmetry-related reflections and/or repeated measurements of the same reflection display specific variations in the substructure amplitude and therefore give rise to intensity differences for which adequate correction cannot be made by multiplicative (scale) factors. Provided that such symmetry-breaking effects in the substructure can be modelled and refined by a set of parameters in real space (*e.g.* coordinates of atomic positions, occupancy factors, atomic scattering factors *etc*.) they can become a source of phase information (Fig. 4 ▶). This requires a paradigm shift in the data-processing strategy, since the usual separation of the data-merging and phasing steps is abandoned. The data are kept unmerged down to the Harker construction, where the symmetry-breaking is explicitly modelled and refined and becomes a source of supplementary phase information. Data merging is effectively carried out on the complex plane, *i.e.* through the generalized Harker construction: from all the data items *F_j_*(*h*) a single quantity **P**(**h**) is estimated but as a complex value.

## 4. ‘Broken symmetries’ in macromolecular crystallography

### 4.1. Anomalous scattering

**Figure 5:**
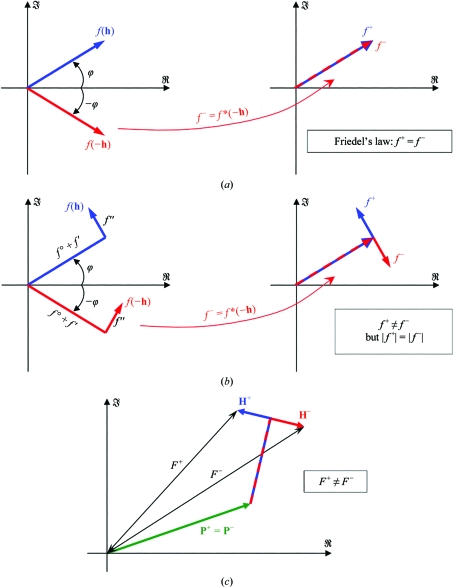
Friedel symmetry-breaking arising from anomalous scattering. (*a*) Structure factor of an atom which has negligible *f*′′: Friedel’s law is satisfied. (*b*) Structure factor of an atom exhibiting anomalous scattering: *f* ^+^ and *f* ^−^ are different, but their moduli are still identical. (*c*) Only when there is a mixture of atoms exhibiting anomalous scattering (substructure **H**) and atoms with negligible anomalous scattering (**P**) will the Friedel opposites have different intensities.

Anomalous scattering is the best-known example of a symmetry-breaking effect (Bijvoet, 1954 ▶) that can be exploited for phase determination (Okaya *et al.*, 1955 ▶; Blow & Rossmann, 1961 ▶; North, 1965 ▶; Matthews, 1966 ▶). The structure factor of a single atom is given by 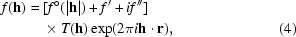
where *T*(**h**) denotes the Debye–Waller factor and all other symbols have their usual meanings. If the anomalous scattering factor *f*′′ is negligibly small, we can write Friedel’s law as 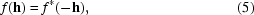
where * denotes complex conjugation and the pair of reflections **h** and −**h** are called Friedel opposites. Let us denote *f* ^+^(**h**) = *f*(**h**) and *f* ^−^(**h**) = f*(−**h**), so that Friedel’s law is written as 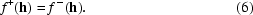
Anomalous scattering breaks the Friedel (or Laue group) equivalence between the reflections **h** and −**h** (Fig. 5 ▶) and can therefore give rise to measurable intensity differences [except for centric reflections, where *f*(**h**) = *f*(−**h**) even in the presence of anomalous scattering]. Again, it is of importance that only the atoms of the substructure are significantly affected by anomalous scattering. If all atoms display similar anomalous scattering, then it is impossible to generate phase information through this effect^1^. In the case of anomalous scattering, we can therefore write 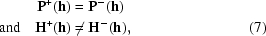
where **P** ^+^(**h**) = **P**(**h**), **P** ^−^(**h**) = P*(−**h**), **H** ^+^(**h**) = **H**(**h**) and **H** ^−^(**h**) = H*(−**h**). Thus, the Harker construction is actually set up with **H** ^+^(**h**) and **H** ^−^(**h**) as the centres of the circles whose radii correspond to the experimentally observed *F*(**h**) and *F*(−**h**) data (Blow & Rossmann, 1961 ▶; North, 1965 ▶).

It is important to note that the Friedel symmetry-breaking effect of anomalous scattering is distinct from its wavelength-dependence, which was mentioned earlier, although in the MAD method both effects are exploited for phase determination. In contrast, in a single-wavelength anomalous dispersion (SAD) experiment only one data set is recorded from the same sample at a single wavelength. The data are left partially unmerged in the sense that Friedel opposites are kept separate. More specifically, the reflections are merged according to the crystal point group, not its Laue group.

### 4.2. Site-specific radiation damage

A number of case studies have established that X-ray-induced damage to crystalline protein samples initially occurs at discrete well localized sites (Burmeister, 2000 ▶; Ravelli & McSweeney, 2000 ▶; Weik *et al.*, 2000 ▶), leading to the breakage of disulfide bonds, the decarboxylation of acidic residues, the loss of hydroxyl groups from tyrosines and the loss of methylthio groups from methionines. Heavier atoms such as selenium in selenosubstituted proteins (Rice *et al.*, 2000 ▶; Ravelli *et al.*, 2005 ▶), bromine in brominated nucleic acids (Ennifar *et al.*, 2002 ▶; Ravelli *et al.*, 2003 ▶; Schiltz *et al.*, 2004 ▶), metals in metalloproteins (Penner-Hahn *et al.*, 1989 ▶; Schlichting *et al.*, 2000 ▶; Berglund *et al.*, 2002 ▶; Yano *et al.*, 2005 ▶) and iodine (Evans *et al.*, 2003 ▶; Zwart *et al.*, 2004 ▶) and mercury (Ramagopal *et al.*, 2005 ▶) in isomorphous derivatives often exhibit a particularly pronounced sensitivity to X-ray damage.

The structure factor of a radiation-sensitive atom changes continuously with time or, more precisely, as a function of the X-­ray dose *d*. In most cases, site-specific radiation damage simply leads to an increased disorder of the atom so that we can write its structure factor as 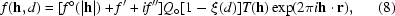
where 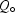 is the zero-dose occupancy of the atom and ξ(*d*) is a continuous function of *d* that varies between the values ξ(0) = 0 and ξ(∞) = 1.

In the presence of site-specific radiation damage, symmetry-related reflections or repeated measurements of the same reflection that are recorded at different X-ray doses will no longer be equivalent since they pertain to different stages of the radiation-induced changes in the substructure. When these data are kept unmerged, and provided that the site-specific modulations of the substructure [*i.e.* the function ξ(*d*) for each atom of the substructure] can be modelled, it is possible to generate phase information from the observed intensity differences *via* the generalized Harker construction.

**Figure 6:**
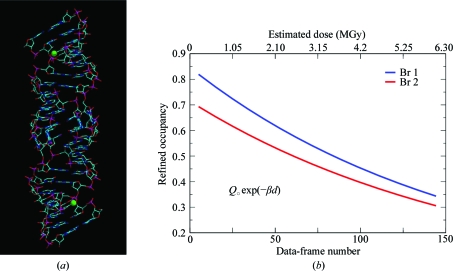
(*a*) Structure of the Br-DIS molecule. Br-DIS is a 23-nucleotide RNA fragment corresponding to the dimerization-initiation site (DIS) of HIV-1(Lai) genomic RNA, with 5-bromouridine substituted for uridine at position 3 (Ennifar *et al.*, 2002 ▶). (*b*) X-ray-induced decay of the Br atoms. The occupancy factors of the Br atoms were refined against unmerged data by applying an exponential decay model [equation (9); Schiltz *et al.*, 2004 ▶]. The rest of the structure remained remarkably stable during the X-­ray data collection. This was therefore an almost ideal example of a case in which the sub­structure (**H**), here consisting of the two Br atoms, undergoes a continuous change during the X-­ray data collection, whereas the remainder of the structure (**P**) remains nearly unchanged. The differential modulation of **H** and **P** gave rise to measurable intensity differences between symmetry-related reflections that were measured at different X-ray doses.

**Figure 7:**
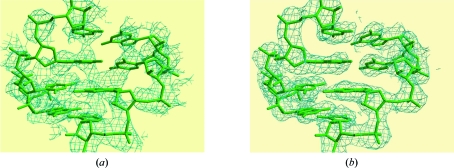
Phasing from unmerged data in the case of Br-DIS (Schiltz *et al.*, 2004 ▶). Views of the electron-density maps obtained for Br-DIS to 2.46 Å resolution after phasing with *SHARP* and solvent flattening. (*a*) Standard SAD phasing on a merged data set. (*b*) A substantial improvement of the SAD phases was obtained by using unmerged data and modelling the X-ray-induced decay of the Br sites. The refined structural model of the Br-DIS molecule is superimposed on the maps.

In the case of the crystal structure determination of Br-DIS, a brominated 23-nucleotide RNA fragment, standard three-wavelength MAD phasing was unsuccessful because of rapid X-­ray-induced debromination (Ennifar *et al.*, 2002 ▶). We demonstrated that a substantial enhancement of the phasing power was achieved by modelling the site-specific changes in a continuous dose-dependent fashion (Fig. 6 ▶) and by keeping the diffraction data unmerged (Schiltz *et al.*, 2004 ▶). The evolution of site-specific radiation damage was explicitly modelled in real space through a very simple exponential decay model with only one refineable parameter, β, for each of the two independent Br atoms, 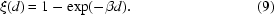
In this example, exploiting the symmetry-breaking effects of site-specific radiation damage enabled the successful phasing of a previously resistant problem (Fig. 7 ▶).

Other situations can arise in which the positions and/or the scattering factors of the substructure atoms also vary as a function of X-ray dose. More elaborate models that are appropriate for such cases have been discussed in Schiltz & Bricogne (2007 ▶).

### 4.3. Polarization anisotropy of anomalous scattering (AAS)

#### 4.3.1. The physical origin of AAS

**Figure 8:**
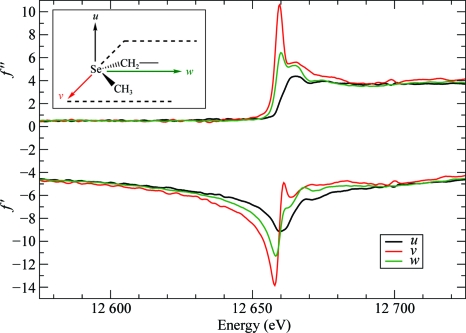
Anomalous scattering factors *f*′ and *f*′′ for Se in selenomethionine residues. The curves represent the anomalous scattering factors when the polarization direction of the incident X-ray beam is aligned with one of the molecular principal directions in a C—Se—C moiety. Black curves: along direction *u* (perpendicular to the plane containing the C—Se—C bonds). Green curves: along direction *w* (bisecting the C—Se—C angle). Red curves: along direction *v* (perpendicular to *u* and *w*). Data from Bricogne *et al.* (2005 ▶).

The anomalous scattering terms for isolated atoms are scalars but, generally, chemical bonding and the symmetry of the atom’s environment induce a directional dependence of *f*′ and *f*′′ on the direction of linear polarization of the X-­ray beam. This anisotropy of anomalous scattering (AAS) is significant in the near-edge region of absorption maxima, as has been experimentally demonstrated in numerous investigations on inorganic and small-molecule com­pounds (Templeton & Templeton, 1982 ▶, 1988 ▶, 1995 ▶; Kirfel *et al.*, 1991 ▶; Dmitrienko, 1983 ▶; Dmitrienko *et al.*, 2005 ▶). AAS has also been observed in selenated proteins (Hendrickson *et al.*, 1990 ▶; Fanchon & Hendrickson, 1990 ▶; Bricogne *et al.*, 2005 ▶; Schiltz & Bricogne, 2008 ▶), in metalloproteins (Hendrickson *et al.*, 1988 ▶) and in brominated nucleotides (Bricogne *et al.*, 2005 ▶; Sanishvili *et al.*, 2007 ▶; Oliéric *et al.*, 2007 ▶). AAS arises from resonant transitions between the core electrons and antibonding valence molecular orbitals that are rendered nonspherically symmetric by the chemical bonding of the absorbing atom. The anomalous scattering thus depends on the relative orientation of the electric field vector of the incident X-ray beam (the polarization direction) with respect to these molecular orbitals. This is illustrated in Figs. 8 ▶ and 9 ▶, respectively, for Se in selenomethionine and Br in brominated nucleotides, which are the two most important anomalous scatterers used for phasing purposes in macromolecular crystallography. The variations in *f*′ and *f*′′ as a function of molecular orientation are very substantial in the near-edge region and are of the same order of magnitude as those that can be obtained by changing the wavelength of the incident beam in a MAD experiment.

In brominated nucleotides, the white line arises through a resonant transition of a 1*s* core electron to an empty antibonding σ* molecular orbital, which is a linear combination of mainly the C 2*sp*_*z*_^2^ and Br 4*p*_*z*_ orbitals (*z* being oriented along the C—Br bond) and therefore has a pronounced *p*_*z*_ symmetry (Sanishvili *et al.*, 2007 ▶). In accordance, in the experimental absorption spectra the white line is observed to be most pronounced along the direction parallel to the C—Br bond, whereas it completely disappears when the polarization vector of the X-ray beam is perpendicular to the C—Br bond. Concomitantly, a large shift of more than 7 eV in the energy position of the minimum (the so-called *inflection point*) is observed in the *f*′ spectra when the direction of polarization is changed from parallel to perpendicular to the C—Br bond.

#### 4.3.2. AAS-induced symmetry-breaking

Synchrotron X-­rays are linearly polarized in the plane defined by the orbit of the electron beam, *i.e.* in the horizontal plane. In principle, the effects of AAS can be revealed by variations in the near-edge absorption spectra and by variations in the diffracted intensities that occur upon changing the orientation of the crystal with respect to the direction of polarization of the X-­ray beam. However, at protein crystallography beamlines a single rotation (spindle) axis is usually employed and this axis is almost universally horizontally oriented, *i.e.* exactly parallel to the direction of polarization of the X-ray beam. Thus, as the crystal is rotated during a data collection the direction of polarization of the incident beam does not change with respect to the crystal. Nevertheless, the polarization of the incident beam can break the crystal symmetry. Since symmetry-related anomalously scattering atoms may experience the incident electric field under different polarization orientations, they are not necessarily equivalent any longer as far as their scattering amplitudes are concerned.

**Figure 10:**
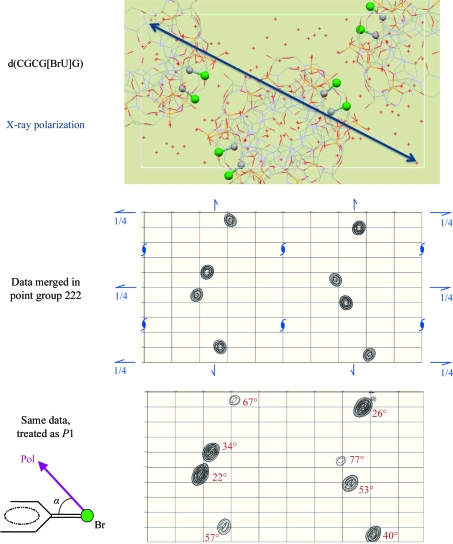
AAS-induced symmetry-breaking in crystals of d(CGCG[BrU]G) (Sanishvili *et al.*, 2007 ▶; Schiltz & Bricogne, 2008 ▶). The crystal packing of d(CGCG[BrU]G) molecules viewed down the crystal *c* axis is displayed in the upper picture. The origin is located in the upper left corner and the *a* axis is along the vertical direction. The eight C—Br moieties in the unit cell are shown, with the Br atoms highlighted as green spheres. Owing to the orientation of the helical DNA duplexes in the crystal, all C—Br bonds are oriented almost perpendicular to [001]. Also displayed is the in-plane component of the X-ray polarization direction, which remained fixed during the whole data collection. The middle picture represents an anomalous difference Fourier map computed from data collected at the Br *K* edge after merging in point group 222. The symmetry elements of the crystal space group *P*2_1_2_1_2_1_ are displayed in blue. The lower map was computed from the same data merged in point group 1, *i.e.* not imposing any symmetry. The figures printed in red next to each peak indicate the angle between the direction of X-ray polarization and the C—Br bond direction of the corresponding Br site.

The symmetry-breaking effects of AAS were cogently demonstrated in the case of a brominated Z-DNA duplex d(CGCG[BrU]G) (Sanishvili *et al.*, 2007 ▶; Schiltz & Bricogne, 2008 ▶; Fig. 10 ▶). X-ray diffraction data were recorded from a single cryocooled crystal at a wavelength corresponding to the position of the Br *K*-edge white line in brominated nucleotides. The data were recorded using a single scan axis oriented parallel to the direction of polarization of the X-ray beam. Hence, during the data collection the direction of polarization remained constant with respect to the crystal and thus also with respect to the C—Br bonds. However, the various symmetry-related C—Br bonds ‘saw’ the polarization direction of the incident beam from different relative orientations and thus gave rise to different effective anomalous scattering factors. The Br atoms with C—Br bonds nearly aligned with the polarization direction exhibited a strong white line (large *f*′′), but this was not the case for symmetry-related Br atoms, where the C—Br bonds were oriented more closely perpendicular to the polarization direction. In a sense, the symmetry operations acted as an ‘internal’ gonio­meter allowing the AAS properties of symmetry-related sites to be sampled at different orientations, even though the orientation of the crystal with respect to the polarization direction remained fixed. In an anomalous difference Fourier map computed with the data merged in point group 1 (*i.e. not* imposing any symmetry), symmetry-related Br sites displayed widely differing peak heights. A clear correlation can be established between the height of each peak and the angle between the corresponding C—Br bond direction and the direction of X-ray polarization.

Similar AAS-induced symmetry-breaking effects were also observed in selenated protein crystals (Schiltz & Bricogne, 2008 ▶).

When reflection data are merged in a certain point or Laue group one actually imposes a symmetry on the crystal structure, so that any genuine intensity differences between symmetry-related reflections are averaged out. Therefore, the widespread practice of merging data prior to phasing com­pletely scrambles the effects of AAS and this may explain why conventional SAD or MAD phasing strategies have not sub­stantially suffered from ignoring AAS altogether. On the other hand, if the data are kept unmerged the intensity differences in symmetry-related reflections can be exploited to model the AAS of anomalously scattering atoms and to generate phase information. Since data sets are usually recorded with a certain degree of redundancy, this additional phase information essentially comes ‘for free’. This can be of particular use in resolving the phase ambiguity in SAD experiments. In Schiltz & Bricogne (2008 ▶), examples were presented of improvements in the quality of phases which were typically of the same order of magnitude as those obtained in a conventional approach by adding a second-wavelength data set to a SAD experiment. Thus, the exploitation of AAS can give access to two-wavelength map quality with single-wavelength measurements. Such a gain is particularly significant, since radiation damage can frequently pre­clude the collection of a second-wavelength data set.

While AAS has been extensively studied in physical, in­organic and small-molecule crystallography, the existence of this phenomenon has until recently remained relatively obscure to protein crystallographers (with the notable exception of Fanchon & Hendrickson, 1990 ▶). Because the details of AAS have not always been well understood, a widely held mis­conception states that AAS in protein crystals is only observable if the molecular groups which surround the anomalously scattering atoms are all aligned in the crystal. A related fallacy is the belief that AAS in protein crystals of high symmetry and/or containing a large number of anomalously scattering atoms in the asymmetric unit would ‘average out’ to isotropy. This is indeed the case for linear X-ray dichroism (as, for example, observed in polarized absorption spectra) and birefringence, which are global (macroscopic) consequences of AAS and which follow the point-group symmetry of the crystal (Bricogne *et al.*, 2005 ▶; Sanishvili *et al.*, 2007 ▶). There can thus be no dichroism in cubic crystals (except for higher order effects, which are usually weak). However, this is clearly not the case for AAS effects in *diffraction*, which are microscopic (local) effects to which each individual atom contributes with its own phase shift. In Schiltz & Bricogne (2008 ▶), we reported substantial AAS effects in the diffracted intensities from crystals of selenomethionine phosphopantetheine adenylyltransferase (PPAT; Izard & Geerlof, 1999 ▶), which crystallizes in the cubic space group *I*23 and contains 384 ordered seleno­methionine groups in the unit cell.

#### 4.3.3. Modelling AAS in macromolecular crystallography

The simplest model for AAS uses ‘symmetry-unrolled’ anomalous scattering factors (Schiltz & Bricogne, 2008 ▶) where for each anomalously scattering atom *in the unit cell* individual anomalous scattering factors *f*′ and *f*′′ are defined and can be freely refined. This model is only valid if all reflections have been recorded with the same polarization direction and therefore applies to the particular, but not uncommon, case in which a data set has been collected by rotating about a horizontal spindle axis. It should be noted that this model is not equivalent to refining all the substructure atoms in space group *P*1. Only the anomalous scattering factors of symmetry-related atoms are refined individually; the positional and thermal parameters are constrained to obey the space-group symmetry, *i.e.* the symmetry-breaking effects are assumed to only originate from the anomalous scattering properties.

In a more general model for AAS the anomalous scattering properties of an atom are described by a second-rank tensor **f**, represented by a symmetric 3 × 3 matrix with complex-valued entries (Templeton & Templeton, 1982 ▶; Dmitrienko, 1983 ▶), 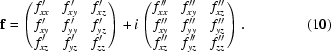
The AAS tensors of two sites *s* and *k* which are symmetry-related through the space-group operation (**R**_*g*_, **t** *_g_*) (such that **r** *_k_* = **R**_*g*_ **r** *_s_* = **t** *_g_*) are related by a similarity transformation involving the rotation operator **R**_*g*_ (Dmitrienko, 1983 ▶), 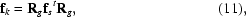
where the left superscript *t* stands for matrix transposition.

We have shown in Schiltz & Bricogne (2008 ▶) that in the context of macromolecular crystallography the anomalous scattering factor of an atom that displays AAS can be approximated by 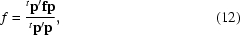
where it is assumed that the incident beam is completely linearly polarized along a direction given by the unit vector **p**. The unit vector **p**′ is obtained by projecting **p** onto a plane perpendicular to the scattered-beam direction and corresponds to the direction of linear polarization of the diffracted beam in the absence of AAS (Schiltz & Bricogne, 2008 ▶, 2009 ▶). Unless the polarization direction **p** is oriented along a symmetry axis, the similarity transformation (11) does not in general give rise to identical scattering factors *f* for symmetry-related sites. The symmetry-breaking effects of AAS can thus be properly modelled by adopting a tensor description for the anomalous scattering factors.

## 5. Implementation

The models for site-specific radiation damage and AAS out­lined in the previous sections have been implemented in the heavy-atom refinement and phasing program *SHARP* (de La Fortelle & Bricogne, 1997 ▶; Bricogne *et al.*, 2003 ▶). The program has been extended for the use of unmerged data and is able to read and processes X-ray dose information for each reflection measurement as well as goniometric information in various forms (Schiltz *et al.*, 200; Schiltz & Bricogne, 2009 ▶). By applying the generalized Harker construction, symmetry-related reflections and repeated measurements of the same reflection can be used, together with data recorded at another wavelength and/or from another heavy-atom derivative or native.

Since the intensity differences between symmetry-related reflections are usually not very large (of the same order of magnitude as Friedel differences), data scaling can be carried out along conventional lines, *i.e.* by minimizing the disagreement between symmetry-related reflections in the crystal Laue group. The parameters of the substructure atoms are usually first refined in conventional mode, *i.e.* without modelling site-specific radiation damage or AAS. Once the refinement of the positional parameters of the substructure atoms has converged, it is possible to switch on the refinement of dose-dependent or AAS parameters.

The modelling and parametrization of non-isomorphism in the case of data affected by site-specific radiation damage or AAS is significantly more complex than for standard cases. The error model that is currently implemented in *SHARP* assumes that the effects of all sources of non-isomorphism are uncorrelated between different observations of a given reflection (de La Fortelle & Bricogne, 1997 ▶; Bricogne *et al.*, 2003 ▶). In essence, a diagonal approximation is used for the non-isomorphism covariance matrix. Such an approximation may not always be entirely justified since non-isomorphism can be correlated across observations that have been recorded under similar geometric conditions (Bricogne *et al.*, 2003 ▶). For a more general treatment it will be necessary to resort to multivariate likelihood functions which are capable of accommodating adequate patterns of covariances between the various observations (Bricogne, 2000 ▶; Pannu *et al.*, 2003 ▶). The implementation of these functions in *SHARP* is currently under way.

## 6. Discussion and conclusion

Although anomalous scattering, site-specific radiation damage and AAS in the substructure are different phenomena with different physical origins, they have the common property of inducing intensity differences between symmetry-related reflections in a diffraction experiment. Provided that these effects are included in a parametrized model of the sub­structure, they can become a source of phase information. With the current practice of recording diffraction data using the single-axis rotation method, it is almost always the case that data sets with a certain degree of redundancy (multiplicity) are collected. Redundancy is often achieved before completeness, *i.e.* several symmetry-equivalent observations of certain reflections are recorded while for other reflections no observations have yet been measured. Thus, redundancy is usually a byproduct of striving to collect a complete data set. In this sense, the additional phase information that may be contained in the intensity differences between symmetry-related reflections essentially comes ‘for free’. Since overall radiation damage is in many cases the main limiting factor in the amount of data that can be collected from a single sample for the purpose of experimental phasing, it is of the utmost importance to be able to derive the maximum amount of phase information from this limited amount of data. A current limitation in the implementation of these methods is the approximate treatment of correlated sources of non-isomorphism.

Although in many cases the standard anomalous signal generated through Bijvoet differences is likely to be the main source of phase information, this can be supplemented by exploiting the symmetry-breaking effects in unmerged data as described above. In particular cases, such as that of the brominated RNA fragment described earlier, site-specific and overall radiation damage evolve on significantly different timescales. The former can then become a very significant source of phase information to complement the anomalous phasing signal. However, such favourable cases are rather atypical. In many ‘real-life’ situations overall radiation damage unfolds at a rate that is not significantly different in comparison to the evolution of site-specific radiation damage or in comparison to the total time that is required to record a complete data set. In such cases the quality of the phases will ultimately be limited by the effects of overall radiation damage, although the proper modelling and exploitation of site-specific radiation damage can still yield a noticeable improvement of phases, as was for instance the case in the structure determination of the PP2A phosphatase activator Ypa2 (Leulliot *et al.*, 2006 ▶; Schiltz & Bricogne, 2007 ▶).

The general question then arises of how to design a data-collection strategy that enables the optimal exploitation of the various possible sources of phase information when the lifetime of the crystal is limited. Crystals can be intentionally misaligned in order to maximize the AAS-induced inequivalence between symmetry-related reflections. However, since the standard anomalous signal is the most important source of phase information, the reduction of systematic errors in Bijvoet intensity differences is of prime importance. In crystals belonging to high-symmetry space groups, Bijvoet pairs are usually recorded in close temporal proximity, even if the crystal is not specially aligned. In such cases there will always be some symmetries that are broken by AAS and these effects can then be exploited for additional phase information. For crystals of lower symmetry the deliberate alignment of a symmetry axis along the spindle allows the simul­taneous recording of Bijvoet pairs. However, if the spindle is oriented horizontally (*i.e.* along the direction of linear polarization of the synchrotron beam) such a geometry will partly or fully neutralize the symmetry-breaking effects of AAS. A more ideal geometry, which would minimize systematic errors in Bijvoet intensity differences by *aligning* a symmetry axis *with respect to the spindle axis*, while at the same time maximizing the symmetry-breaking effects of AAS by *misaligning* the symmetry axis *with respect to the direction of X-ray polarization*, can be achieved with multi-axis goniometers, where the scan axis is not con­strained to be aligned with the X-ray polarization direction. Thus, with the future use of goniometers with a vertical scan axis designed for the purposes of gaining mechanical stability in the handling of microcrystals, the effects of AAS will become truly ubiquitous in all data sets collected at an absorption edge of a covalently bonded anomalous scatterer and their proper treatment in experimental phasing will be imperative if a major waste of phase information is to be avoided.

## Figures and Tables

**Figure 9:**
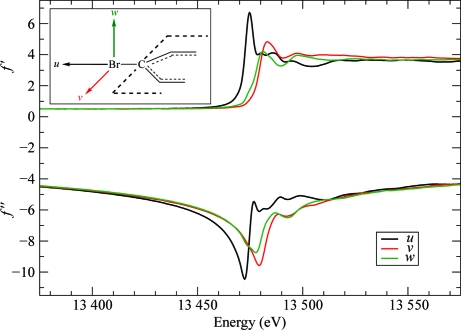
Anomalous scattering factors *f*′ and *f*′′ for Br in brominated nucleotides. The curves represent the anomalous scattering factors when the polarization direction of the incident X-ray beam is aligned with one of the molecular principal directions in a brominated nucleobase. Black curves: along direction *u* (parallel to the C—Br bond). Red curves: along direction *v* (perpendicular to the C—Br bond and parallel to the plane of the nucleobase ring). Green curves: along direction *w* (perpendicular to the nucleobase ring). Data from Sanishvili *et al.* (2007 ▶).

## Appendix A The generalized Harker construction

Even in the absence of symmetry-breaking effects, the com­plex structure factors of symmetry-equivalent reflections are, in general, not identical; only their moduli are. Some care therefore has to be exercised when setting up the Harker construction with symmetry-related reflections. All complex structure factors have to be transformed to a common unique representative. The situation is similar to that encountered in building up the Harker construction with Friedel pairs in the case of anomalous scattering. In that case, the Harker con­struction is actually set up with **F**(**h**) and **F***(−**h**), *i.e.* **F**(−**h**) is reflected through the real axis on the complex plane (Blow & Rossmann, 1961 ▶; North, 1965 ▶).

Let  denote the space group of the crystal. The operation of an element *g* of  will be written as

Reflections **h** *_u_* and **h** *_v_* are symmetry-related if

The set of all reflections **h** *_v_* which satisfy (14) is called the *orbit* of **h** *_u_*. It is customary to select in each orbit one reflection, simply denoted by **h**, as a *unique* representative. The set of all unique reflections constitutes an asymmetric unit in reciprocal space. We can then simply label symmetry-related reflections by *g*,

In the absence of symmetry-breaking effects, the structure factors of symmetry-equivalent reflections are related by (Waser, 1955 ▶)

If the symmetry-related reflections **h** and **h**_*g*_ are to be used on the same Harker construction, it is thus necessary to rotate **F**(**h**_*g*_) back to the phase angle of **F**(**h**), *i.e.* undo the phase shift exp(−2π*i* **h**·**t** *_g_*) and therefore define

In addition, we also want to include the Friedel opposite −**h** and its symmetry-related reflections on the Harker construction. For acentric reflections, **h** and −**h** form distinct orbits, which we label by σ = ±, respectively. We therefore extend our definition to

In the absence of symmetry-breaking effects, all  defined in this way will be identical for all  and for any sign σ = {+, −} (strict symmetry equivalence, including Friedel equivalence). If we assume, as before, that the unknown part of the structure factor **P**(**h**) is unaffected by any symmetry-breaking effects, we can write

Isotropic anomalous scattering in the substructure breaks the Friedel symmetry of acentric reflections, so that

but otherwise the symmetry equivalence is preserved,

In the presence of AAS or site-specific radiation damage, all  are potentially different for the various  and signs σ = {+, −}.

In conclusion then, the generalized Harker construction is set up with circles whose radii correspond to observed amplitudes *F*_σ,*g*_(**h**) of all the reflections which are symmetry-related (including Friedel symmetry) to **h**. Each circle is centred at a position  given by (18). In an ideal (error-free) case, all the circles will then intersect at the point **P**(**h**).

## Footnotes

1 As an example, in the case of elemental Se in its helical chiral (noncentrosymmetric) crystal structure there is no difference in intensity between Friedel opposites (Fig. 5 ▶). For further discussion, see Flack & Shmueli (2007 ▶).
